# Medical Association of Georgia

**Published:** 1860-05

**Authors:** 


### Medical Association of Georgia.

As will be perceived from the abstract of the proceedings, published in this number of the Journal, this body determined at its recent meeting to assemble again in Atlanta, next year. As stated before that body, we are pleased at all times to entertain our professional brethren, as members of this Association, whenever they may select this point as the place for their meeting.

The Association also adopted a resolution notifying the members that at the next meeting, the question of selecting a permanent location for the Association would be presented and decided upon, as also the designation of the point for that location, in the event of their determination to make it a fixture. We do not propose to discuss the subject at present, but will merely state now, that we most earnestly desire that policy to be adopted, which will be most likely to result in the prosperity of the Association.

